# Successful Treatment of *Klebsiella pneumoniae* NDM Sepsis and Intestinal Decolonization with Ceftazidime/Avibactam Plus Aztreonam Combination in a Patient with TTP Complicated by SARS-CoV-2 Nosocomial Infection

**DOI:** 10.3390/medicina57050424

**Published:** 2021-04-28

**Authors:** Francesco Perrotta, Marco Paolo Perrini

**Affiliations:** 1 Department of Anesthesia and Intensive Care Unit, IRCCS Casa Sollievo della Sofferenza, Viale Cappuccini, 1, 71013 San Giovanni Rotondo, FG, Italy; francperr72@yahoo.it; 2 Department of Anesthesia and Intensive Care Unit, Università degli Studi di Foggia, Azienda Ospedaliero Universitaria Ospedali Riuniti di Foggia, 1, 71122 Viale Pinto, FG, Italy

**Keywords:** multidrug-resistant bacteria, *Klebsiella* NDM, ceftazidime-avibactam

## Abstract

Carbapenem-resistant *Enterobacteriaceae* (CRE) are a serious public health threat. Infections due to these organisms are associated with significant morbidity and mortality. Among them, metallo-β-lactamases (MBLs)-producing *Klebsiella pneumoniae* are of global concern today. The ceftazidime/avibactam combination and the ceftazidime/avibactam + aztreonam combination currently represent the most promising antibiotic strategies to stave off these kinds of infections. We describe the case of a patient affected by thrombotic thrombocytopenic purpura (TTP) admitted in our ICU after developing a hospital-acquired SARS-CoV-2 interstitial pneumonia during his stay in the hematology department. His medical conditions during his ICU stay were further complicated by a *K. Pneumoniae* NDM sepsis. To our knowledge, the patient had no risk factors for multidrug-resistant bacteria exposure or contamination during his stay in the hematology department. During his stay in the ICU, we treated the sepsis with a combination therapy of ceftazidime/avibactam + aztreonam. The therapy solved his septic state, allowing for a progressive improvement in his general condition. Moreover, we noticed that the negativization of the hemocultures was also associated to a decontamination of his known rectal colonization. The ceftazidime/avibactam + aztreonam treatment could not only be a valid therapeutic option for these kinds of infections, but it could also be considered as a useful tool in selected patients’ intestinal decolonizations.

## 1. Introduction

The prevalence of multidrug-resistant organisms (MDROs), a major public health threat, continues to increase on a global level and is associated with significant morbidity and mortality.

Phenotypic resistance to carbapenems is typically caused by the β-lactamase activity combined with structural mutations and the production of carbapenemases, enzymes that hydrolyze carbapenem antibiotics.

Other mechanisms associated with carbapenem resistance in Gram-negative bacteria (GNB) include drug efflux pumps and alterations in penicillin-binding proteins.

These characteristics are generally located on mobile genetic elements (MGE) or linked to the hyperproduction of enzymes from inducible or derepressed chromosomal genes [1,2].

In a single-center longitudinal study in long-term acute care hospital, patients who were colonized with carbapenemase-producing *Klebsiella pneumoniae* (KPC-Kp) were studied by Shimasaki et al., who found that carbapenem use was associated with an increased hazard for high relative abundance of KPC-Kp in the gut microbiota. Additionally, high relative abundance of KPC-Kp was associated with KPC-Kp bacteremia [3].

As already known, bacteriemia can rapidly evolve into sepsis and septic shock, especially if it occurs in debilitated patients affected by systemic diseases and needing intensive care unit treatments.

In Italy, the national surveillance carried out in the period 2014–2017 reported 7632 carbapenemase-producing *Enterobacteriaceae* blood stream infections (CPE BSI) from all Italian regions and autonomous provinces. 

Most of the cases (7490, 98.1%) were due to *K. pneumoniae*, while *E. coli* was reported in only 142 (1.9%) cases.

A carbapenemase enzyme identified in the CPE strains and isolated from BSI was reported in 60.4% (4612/7632) of cases.

In most cases, the enzyme reported was *Klebsiella pneumoniae* carbapenemase (in 95.2% of *K. pneumoniae* and 81.4% of *E. coli*). Metallo-beta-lactamases (MBL) were reported in 2.1% of cases, and carbapenem-hydrolyzing oxacillinase-48 (OXA-48) in 1.2%. Associations between MBL and KPC (0.9%) or MBL and OXA-48 (0.3%) were also identified in rare cases.

MBL-detected genes were mainly Verona integron-encoded metallo-beta-lactamase (VIM) (65/86, 75.6%) followed by New Delhi metallo-beta-lactamase (NDM) (21/86, 24.4%). NDM was reported only in the years 2016–2017, mainly in *K. pneumoniae* (20/21, 95.2%), and often in association with OXA-48 [4].

The high incidence of KPC-CPE in Italy favors the use of ceftazidime/avibactam (CAZ/AVI), a combination of a well-established β-lactam antibiotic, ceftazidime, with a novel β-lactamase inhibitor, avibactam, for treatment of serious infections caused by resistant Gram-negative pathogens. Ceftazidime/avibactam is active against OXA-48- and KPC-producing *Enterobacteriaceae*, but not NDM- or VIM-producing *Enterobacteriaceae*, and would offer a partial solution to treat infections due to XDR or PDR Gram-negative bacteria [5].

Metallo-β-lactamase (NDM) is the most recently discovered carbapenemase capable of hydrolyzing almost all β-lactams present in Gram-negative pathogens produced mainly by *K. pneumoniae* and *Escherichia coli*, and is responsible for hospital and acquired infections in community [6].

In vitro data support the use of aztreonam (ATM) with ceftazidime/avibactam (CAZ/AVI) combination, but clinical studies are lacking. In a recent study, the CAZ/AVI + ATM combination offered a therapeutic advantage over other antibiotics for patients with BSI due to MBL-producing *Enterobacterales* [7].

We describe the successful treatment of a patient with the ceftazidime/avibactam + aztreonam combination below. This was effective not only in the treatment of sepsis, but also in the intestinal decolonization.

To our knowledge, this is the first case report in literature on intestinal decontamination using the ceftazidime/avibactam + aztreonam combination as treatment for *Klebsiella pneumoniae* NDM sepsis.

## 2. Case Report

On 27 November 2020, during the main peak of the second wave of the SARS-CoV-2 pandemic, a 57-year-old man was admitted to the hematology department of our hospital with a diagnosis of acute TTP.

On admission, the physical examination revealed jaundice and purpura throughout the body; the total Khellaf score was 9.

Laboratory evaluation revealed a total platelet count of 8 × 10^3^/μL, increased levels of total bilirubine (6.2 mg/dL), and serum creatinine (2.2 mg/dL), LDH (1499 mU/mL), with an Adamts 13 value of <0.01. A peripheral blood smear showed numerous schistocytes. Direct and indirect Coombs tests came back negative.

On the basis of these findings, we started plasma exchange therapy (a total of six sessions) together with methylprednisolone at 40 mg twice daily, along with a treatment by the humanized anti-von Willebrand Factor (vWF) nanobody caplacizumab of 10 mg after each plasma exchange session.

Seven days after the beginning of the therapy we observed a complete remission of the TTP.

On day 10 of hospitalization, the patient suddenly developed a fever and dyspnea requiring continuous oxygen support; the chest X-ray showed an image of diffuse bilateral opacities suggestive of an extended interstitial evolving pneumonia, and a nasopharyngeal swab was therefore realized and tested positive for SARS-CoV-2.

The further necessity for non-invasive mechanical ventilation required an ICU hospitalization on 11 December 2021.

Because of the fast-increasing respiratory distress, at day 2 of ICU hospitalization, we proceeded with the sedation, curarization, orotracheal intubation, and connection to mechanical ventilatory support.

Upon admission, the patient’s signs and symptoms and the biological response to TTP were in regression: the total platelets count was 240 × 10^3^/μL, serum creatinine was 1.3 mg/dL, and the level of total bilirubine was 1.5 mg/dL.

A CPE surveillance rectal swab performed on admission in the ICU tested positive for *Klebsiella Pneumoniae* NDM.

The patient had no recent history of hospitalization and no other predisposing risk factors. Thus, a horizontal transfer during the stay in hematology or a rare community acquired colonization were conceivable.

Between day 2 and day 3 of hospitalization in the intensive care unit, the patient conditions worsened: the total platelets count dropped to 5 × 103/μL together with an increase in the serum creatinine, increasing levels of total bilirubine associated to the hemolysis, and Pct levels up to 7.12 ng/mL and WBC up to 1.46 × 10^3^/μL.

The patient had developed a severe septic condition (SOFA score 9) that was causing the exacerbation of the TTP, thus requiring further cycles of plasma exchange.

After performing blood and urine cultures, we started a therapy with CAZ/AVI 1.25 mg q8 h plus AZT 1 gr q8 h for up to 10 days; we decided not to use colistin because of the renal failure.

Both the blood and urine cultures tested positive for KPC NMD with the same antibiogram profile tested in the rectal swab (Table 1).

The patient’s clinical conditions improved soon after the therapy introduction and after 10 days of treatment, the blood culture and rectal swab both tested negative; the resolution of the sepsis and the clinical improvement of the patient’s conditions allowed for a progressive weaning, and finally, extubation at day 28.

The patient was then transferred to the medical ward for post-ICU rehabilitation.

## 3. Discussion

New Delhi metallo-β-lactamase (NDM-1), is one of the most clinically significant carbapenemases. It was first reported in New Delhi, India [6], followed by several clinical cases in the UK, Pakistan, and now around the world [2].

A large outbreak sustained by New Delhi metallo-β-lactamase (NDM)-producing *Enterobacterales* was recently documented in Tuscany, Italy [8].

In 2020, NDM-9-producing *Klebsiella pneumoniae* were isolated in the same geographic area.

A genomic and phylogenetic study suggested the correlation of the 2018–2019 and 2020 strains, with a change from the NDM-1 to NDM-9 carbapenemase variant in the latter [9]. No apparent changes in beta-lactam susceptibility were detected, and in addiction, new mutations in chromosomally encoded genes showed an acquired resistance to tigecycline, fosfomycin, and colistin.

The plasmid-carried bla-NDM gene is a transferable gene and it is capable of extensive rearrangement. This strongly suggests a marked capability for horizontal transfer from a colonized patient and a greater likelihood of occurrence between different bacterial strains.

Aztreonam, a monobactam antibiotic, is active against MBL-producing bacteria, but it is hydrolyzed by Ambler class A beta-lactamases (e.g., ESBL and KPCs) and class C (e.g., AmpC) beta-lactamases [10].

Since many MBL-producing Gram-negative bacteria may simultaneously express beta-lactamases or carbapenemases that could hydrolyze aztreonam, the combination with avibactam is able to inhibit cell wall synthesis in MBL-producing strains despite the presence of other co-carried beta-lactamases.

Aztreonam–avibactam showed a potent in-vitro activity against ESBL, class C β-lactamase, MBL, and KPC-producing strains with an activity 10 times that of aztreonam alone.

However, limited activity has been shown against *A. baumannii* or *P. aeruginosa* compared with aztreonam alone [11].

The potential for the use of CZA in combination with MEM, AMK, AZT, COL, and FOS against MDR *P. aeruginosa* and carbapenemase-producing *K. pneumoniae* was investigated in a recent publication [12]. Unfortunately, this study was not focused on MBL-producing bacteria, and surely, further research is merited to clarify the mechanisms of enhanced activity between CZA with MEM and other antibiotics together with further tests in clinical settings.

A phase III clinical trial to compare aztreonam–avibactam (with or without metronidazole) with meropenem (with or without colistin) for the treatment of HAP, VAP, and cIAI due to Gram-negative bacteria, for which there are limited or no treatment options, started in March 2018 and will end in 2021 [NCT03329092].

In a 2017 publication, Marshal et al. showed the in vitro efficacy of a unique combination of CAZ/AVI and ATM against most of the 21 representative *Enterobacteriaceae* isolates, with a complex molecular background that included blaIMP, blaNDM, blaOXA-48, blaCTX-M, blaAmpC, and combinations thereof [13].

This opened the way for the most promising new combination, currently available for NDM- or VIM-producing KPC.

From another point of view, key synergies in treating MBL infections especially lie with the action of avibactam.

Avibactam, inhibiting class A, C, and D β-lactamases, is thus supposed to leave ceftazidime and aztreonam not hydrolyzed by MBL, allowing them to act [14].

Another propriety of non-β-lactam β-lactamase inhibitor avibactam, as reported by Asli et al., is the ability to covalently bind to some bacterial PBPs, such as *E. coli* and *H. influenzae* PBP2, PBPs 2 and 3 of *P. aeruginosa* and *S. aureus*, and PBP3 of *S. pneumoniae*. This capacity may explain its moderate antibacterial activity against some bacterial strains and species [15].

Patients like the one reported in our case provide strong evidence that intestinal colonization with KPC-Kp is associated with an increased hazard for high relative abundance of KPC-Kp in the gut microbiota, and additionally, the increased risk of KPC-Kp bacteremia [3].

As is already known, bacteriemia can rapidly evolve into sepsis and septic shock, especially if it occurs in debilitated patients affected by systemic diseases and needing intensive care unit treatments.

The COVID-19 pandemic continues to challenge healthcare systems around the world, and is indeed adding further challenges that we still do not fully understand in the antibiotic-resistance field.

The dramatically increased number of ICU hospitalizations, the direct or indirect role of SARS-CoV-2 in the individual augmented risk for developing superinfections, and the spreading of nosocomial infections requiring antibiotic treatments on such a large scale will be impactful, and precipitate further widespread adverse health outcomes that we should be prepared for.

Like what happened with our patient, a SARS-CoV-2 infection could rapidly lead to the worsening of the previous clinical conditions and the need for ICU hospitalization.

There are many ways beyond interstitial pneumonia—linked to opportunistic infections—in which a COVID-19 infection could rapidly worsen already unstable conditions of a hospitalized patient.

SARS-CoV-2 has linked impaired antigen presentation or acquired immunosuppression with concomitant lymphopenia [16,17], and the macro- and microcirculation alterations associated to the SARS-CoV-2 hypercoagulability [18] can likely increase the risk of bacterial translocation [19], the classic example being the intestinal tract.

Endothelial dysfunctions of the digestive tract were frequently observed in COVID-19 and were associated with different mesenteric infarctions [20] that could explain the gut flora alteration and increased risk of translocations.

These considerations have also been taken into account in a recent French case–cohort study from the multicentric OUTCOMEREA network that showed how the daily hazard rate of ICU BSI in critically ill COVID-19 patients increased, and that this was more frequent seven days after ICU admission [21].

The extended use of immune-modulatory treatments, such as IL-1 or IL-6 receptors or antagonists in critically ill patients affected by SARS-CoV-2, could surely play a role in these findings, as suggested by the French study.

There is an open literature debate about this argument. On the one hand (like in work recently published by the RECOVERY collaborative group [NCT04381936]), some studies are showing the non-negligible benefits associated with dexamethasone without significant increased risk of developing secondary infections compared with standards of care. Similar findings about the risk of secondary infections were also shown in two other recent RCTs [22,23].

On the other hand, some randomized trials of tocilizumab in COVID-19 have so far shown mixed results for 28–83 day mortality, and in a recent randomized-controlled trial [24], there was an increased risk of superinfections, and a similar trend was shown in the BSI.

The last, and more particular point of view in our case, is how interestingly the ceftazidime/avibactam + aztreonam combination therapy used was able to succeed in eradicating the *Klebsiella* NDM strain from the patient’s gut as confirmed by two molecular tests.

The eradication of carbapenemase-producing *Klebsiella* from the gut is still a challenge and a matter of discussion.

Although various efforts have been made with both oral drugs and combined oral and systemic drugs, only partial results have been obtained.

Ceftazidime/avibactam plus aztreonam therapy could be one of the more useful solutions to consider in this field for selected patients.

## 4. Conclusions

The ceftazidime/avibactam + aztreonam treatment could not only be a valid therapeutic option for MBL-producing *Enterobacterales* infections, but it could also be considered as a useful tool in selected patients’ intestinal decolonization.

## Figures and Tables

**Table 1:** Resistance profile of the *K. pneumoniae* NDM strain found in the blood samples.

		*MIC Breakpoint*		
Antibiotics		MIC	MICS	MICR
**Cefepime**	R	≥32	1	4
**Cefotaxime**	R	≥64	1	2
**Ceftazidime**	R	≥64	1	4
**Ceftazidime/Avibactam**	R	≥16	8	8
**Ceftolozane/Tazobactam**	R	≥32	2	2
**Ciprofloxacine**	R	≥4	0.25	0.5
**Colistin**	S	≤0.5	2	2
**Gentamicine**	R	≥16	2	4
**Imipenem**	R	≥16	2	4
**Meropenem**	R	≥16	2	8
**Piperacilline/Tazobactam**	R	≥128	8	16
**Tobramycin**	R	≥−16	2	2
**Trimetoprim/Sulfam**	R	≥320	40	80

## References

[1] Logan L.K., Weinstein R.A. (2017). The epidemiology of Carbapenem-resistant enterobacteriaceae: The impact and evolution of a global menace. J. Infect. Dis..

[2] Nordmann P., Dortet L., Poirel L. (2012). Carbapenem resistance in Enterobacteriaceae: Here is the storm!. Trends Mol. Med..

[3] Shimasaki T., Seekatz A., Bassis C., Rhee Y., Yelin R.D., Fogg L., Dangana T., Cisneros E.C., Weinstein R.A., Okamoto K. (2019). Increased Relative Abundance of Klebsiella pneumoniae Carbapenemase-producing Klebsiella pneumoniae within the Gut Microbiota Is Associated with Risk of Bloodstream Infection in Long-term Acute Care Hospital Patients. Clin. Infect. Dis..

[4] Iacchini S., Sabbatucci M., Gagliotti C., Rossolini G.M., Moro M.L., Iannazzo S., D’Ancona F., Pezzotti P., Pantosti A. (2019). Bloodstream infections due to carbapenemaseproducing Enterobacteriaceae in Italy: Results from nationwide surveillance, 2014 to 2017. Eurosurveillance.

[5] Albiger B., Glasner C., Struelens M.J., Grundmann H., Monnet D.L. (2015). Carbapenemase-producing Enterobacteriaceae in Europe: Assessment by national experts from 38 countries, May 2015. Eurosurveillance.

[6] Yong D., Toleman M.A., Giske C.G., Cho H.S., Sundman K., Lee K., Walsh T.R. (2009). Characterization of a new metallo-β-lactamase gene, bla NDM-1, and a novel erythromycin esterase gene carried on a unique genetic structure in Klebsiella pneumoniae sequence type 14 from India. Antimicrob. Agents Chemother..

[7] Falcone M., Daikos G.L., Tiseo G., Bassoulis D., Giordano C., Galfo V., Leonildi A., Tagliaferri E., Barnini S., Sani S. (2020). Efficacy of Ceftazidime-avibactam Plus Aztreonam in Patients with Bloodstream Infections Caused by Metallo-β-lactamase–Producing Enterobacterales. Clin. Infect. Dis..

[8] Tavoschi L., Forni S., Porretta A., Righi L., Pieralli F., Menichetti F., Falcone M., Gemignani G., Sani S., Vivani P. (2020). Prolonged outbreak of New Delhi metallo-betalactamase-producing carbapenem-resistant Enterobacterales (NDM-CRE), Tuscany, Italy, 2018 to 2019. Eurosurveillance.

[9] Falcone M., Giordano C., Barnini S., Tiseo G., Leonildi A., Malacarne P., Menichetti F., Carattoli A. (2020). Extremely drug-resistant NDM-9-producing ST147 Klebsiella pneumoniae causing infections in Italy, May 2020. Eurosurveillance.

[10] Jean S.-S., Lee W.-S., Lam C., Hsu C.-W., Chen R.-J., Hsueh P.-R. (2015). Carbapenemase-producing Gram-negative bacteria: Current epidemics, antimicrobial susceptibility and treatment options. Future Microbiol..

[11] Bassetti M., Vena A., Castaldo N., Righi E., Peghin M. (2018). New antibiotics for ventilator-associated pneumonia. Curr. Opin. Infect. Dis..

[12] Mikhail S., Singh N.B., Kebriaei R., Rice S.A., Stamper K.C., Castanheira M., Rybak M.J. (2019). Evaluation of the synergy of ceftazidime-avibactam in combination with meropenem, amikacin, aztreonam, colistin, or fosfomycin against well-characterized multidrug-resistant Klebsiella pneumoniae and Pseudomonas aeruginosa. Antimicrob. Agents Chemother..

[13] Marshall S., Hujer A.M., Rojas L.J., Papp-Wallace K.M., Humphries R.M., Spellberg B., Hujer K.M., Marshall E.K., Rudin S.D., Perez F. (2017). Can Ceftazidime-Avibactam and Aztreonam Overcome β-Lactam Resistance Conferred by Metallo-β-Lactamases in *Enterobacteriaceae*?. Antimicrob. Agents Chemother..

[14] Drawz S.M., Papp-Wallace K.M., Bonomo R.A. (2014). New β-lactamase inhibitors: A therapeutic renaissance in an MDR world. Antimicrob. Agents Chemother..

[15] Asli A., Brouillette E., Krause K.M., Nichols W.W., Malouin F. (2016). Distinctive binding of avibactam to penicillin-binding proteins of gram-negative and gram-positive bacteria. Antimicrob. Agents Chemother..

[16] Giamarellos-Bourboulis E.J., Netea M.G., Rovina N., Akinosoglou K., Antoniadou A., Antonakos N., Damoraki G., Gkavogianni T., Adami M.-E., Katsaounou P. (2020). Complex Immune Dysregulation in COVID-19 Patients with Severe Respiratory Failure. Cell Host Microbe.

[17] Terpos E., Ntanasis-Stathopoulos I., Elalamy I., Kastritis E., Sergentanis T.N., Politou M., Psaltopoulou T., Gerotziafas G., Dimopoulos M.A. (2020). Hematological findings and complications of COVID-19. Am. J. Hematol..

[18] Dobesh P.P., Trujillo T.C. (2020). Coagulopathy, Venous Thromboembolism, and Anticoagulation in Patients with COVID-19. Pharmacotherapy.

[19] Cardinale V., Capurso G., Ianiro G., Gasbarrini A., Arcidiacono P.G., Alvaro D. (2020). Intestinal permeability changes with bacterial translocation as key events modulating systemic host immune response to SARS-CoV-2: A working hypothesis. Dig. Liver Dis..

[20] Cheung K.S., Hung I.F., Chan P.P., Lung K.C., Tso E., Liu R., Ng Y.Y., Chu M.Y., Chung T.W., Tam A.R. (2020). Gastrointestinal Manifestations of SARS-CoV-2 Infection and Virus Load in Fecal Samples From a Hong Kong Cohort: Systematic Review and Meta-analysis. Gastroenterology.

[21] Buetti N., Ruckly S., de Montmollin E., Reignier J., Terzi N., Cohen Y., Shiami S., Dupuis C., Timsit J.-F. (2021). COVID-19 increased the risk of ICU-acquired bloodstream infections: A case–cohort study from the multicentric OUTCOMEREA network. Intensive Care Med..

[22] Salvarani C., Dolci G., Massari M., Merlo D.F., Cavuto S., Savoldi L., Bruzzi P., Boni F., Braglia L., Turrà C. (2021). Effect of Tocilizumab vs. Standard Care on Clinical Worsening in Patients Hospitalized with COVID-19 Pneumonia: A Randomized Clinical Trial. JAMA Intern. Med..

[23] Hermine O., Mariette X., Tharaux P.L., Resche-Rigon M., Porcher R., Ravaud P., Bureau S., Dougados M., Tibi A., Azoulay E. (2021). Effect of Tocilizumab vs. Usual Care in Adults Hospitalized with COVID-19 and Moderate or Severe Pneumonia: A Randomized Clinical Trial. JAMA Intern. Med..

[24] Aziz M., Haghbin H., Abu Sitta E., Nawras Y., Fatima R., Sharma S., Lee-Smith W., Duggan J., Kammeyer J.A., Hanrahan J. (2021). Efficacy of tocilizumab in COVID-19: A systematic review and meta-analysis. J. Med. Virol..

